# Nuclear envelope transmembrane proteins involved in genome organization are misregulated in myotonic dystrophy type 1 muscle

**DOI:** 10.3389/fcell.2022.1007331

**Published:** 2023-01-09

**Authors:** Vanessa Todorow, Stefan Hintze, Benedikt Schoser, Peter Meinke

**Affiliations:** Friedrich-Baur-Institute at the Department of Neurology, University Hospital, LMU, Munich, Germany

**Keywords:** nuclear envelope, myotonic dystrophy type 1, genome organization, muscle biopsy, nuclear envelope transmembrane proteins

## Abstract

Myotonic dystrophy type 1 is a multisystemic disorder with predominant muscle and neurological involvement. Despite a well described pathomechanism, which is primarily a global missplicing due to sequestration of RNA-binding proteins, there are still many unsolved questions. One such question is the disease etiology in the different affected tissues. We observed alterations at the nuclear envelope in primary muscle cell cultures before. This led us to reanalyze a published RNA-sequencing dataset of DM1 and control muscle biopsies regarding the misregulation of NE proteins. We could identify several muscle NE protein encoding genes to be misregulated depending on the severity of the muscle phenotype. Among these misregulated genes were NE transmembrane proteins (NETs) involved in nuclear-cytoskeletal coupling as well as genome organization. For selected genes, we could confirm that observed gene-misregulation led to protein expression changes. Furthermore, we investigated if genes known to be under expression-regulation by genome organization NETs were also misregulated in DM1 biopsies, which revealed that misregulation of two NETs alone is likely responsible for differential expression of about 10% of all genes being differentially expressed in DM1. Notably, the majority of NETs identified here to be misregulated in DM1 muscle are mutated in Emery-Dreifuss muscular dystrophy or clinical similar muscular dystrophies, suggesting a broader similarity on the molecular level for muscular dystrophies than anticipated. This shows not only the importance of muscle NETs in muscle health and disease, but also highlights the importance of the NE in DM1 disease progression.

## Introduction

Myotonic dystrophy type 1 (DM1) is clinically characterized by multisystemic involvement with skeletal muscle and brain being the primarily affected organs. Clinical symptoms include myotonia, skeletal muscle weakness and wasting, cardiac arrhythmia, cataracts and insulin resistance, endocrine dysfunction, frontal balding and a shortened lifespan (Udd and Krahe, 2012; Thornton, 2014; Wenninger et al., 2018). An estimated prevalence of about one in 8,000 and the predominant muscle involvement make DM1 one of the most frequent muscular dystrophies in adulthood (Faustino and Cooper, 2003; Wheeler, 2008).

Genetically, DM1 is caused by a pathological CTG-repeat expansion in the 3′UTR of the *DMPK* (dystrophia myotonia protein kinase) gene (Fu et al., 1992). The extended repeat is unstable, up to 35 CTG-repeats are found in healthy individuals, and between 35 and 49 repeats are considered to be a premutation (Udd and Krahe, 2012). The longer the repeat, the more severe the clinical presentation: between 50 and ∼150 repeats usually result in a mild phenotype, a range from ∼100 to ∼1,000 repeats has been identified in patients with classical DM1, and more than 1000 CTG-triplets usually result in congenital DM, the most severe form of the disease. This rough correlation between repeat length and severity of the disease is non-linear (De Antonio et al., 2016), and there are other factors contributing to the clinical presentation. Maternal inheritance results in more severe symptoms than paternal inheritance, which may be due to an increased greater instability of mutant alleles in female meiosis or maternal-biased CpG methylation of the *DMPK* locus (Rakocevic-Stojanovic et al., 2005; Martorell et al., 2007; Barbé et al., 2017). The extended repeats are somatically instable, usually resulting in increase of repeat length during the lifetime of an affected individual and somatic mosaicism (Monckton et al., 1995; Wong et al., 1995). Especially for skeletal muscle it has been shown that repeats can be three- and 25-fold longer than in leukocytes (Thornton et al., 1994; Nakamori et al., 2013).

The mechanisms proposed to contribute to the DM1 phenotype include alternative splicing of several mRNAs (Ho et al., 2005; López-Martínez et al., 2020), altered transcriptional regulation (Ebralidze et al., 2004; Osborne et al., 2009), miRNA misregulation (Rau et al., 2011; Kalsotra et al., 2014; Shen et al., 2020) and inhibited translation (Huichalaf et al., 2010; Meola et al., 2013). The most intensively investigated mechanism is probably alternative splicing, caused by the formation of hairpin structures in the extended CUG-repeat containing *DMPK* RNA transcripts (Napierała and Krzyzosiak, 1997). These secondary structures sequester several RNA-binding proteins, with muscle-blind proteins (MBNL1-3) being the most prominent ones (Fardaei et al., 2001; Fardaei et al., 2002). This results in a nucleoplasmic depletion of MBNL and therefore loss of function. Another splicing factor, CUGBP elav-like family member 1 (CELF1), gets stabilized in parallel by hyperphosphorylation causing a gain of function (Philips et al., 1998; Kuyumcu-Martinez et al., 2007). In total, this leads to a misbalance of splicing and a shift towards an embryonic splicing pattern. Missplicing of a set of muscle-specific genes including *TTN* (titin), *DMD* (dystrophin) (Yamashita et al., 2012), *CLCN1* (chloride voltage-gated channel 1) (Charlet et al., 2002; Mankodi et al., 2002), and *RYR1* (ryanodine receptor 1) (Kimura et al., 2005), among others, can be directly linked to specific DM1 symptoms.

Despite all this information, it is still unclear which mechanism is contributing to which extent, and if yet unknown factors add to the development of this complex disease—especially in the different tissues affected. Intriguingly, alterations to the nuclear envelope (NE) structure and expression changes of NE transmembrane proteins (NETs) have been observed in primary DM1 myoblast and myotube cultures (Hintze et al., 2018; Meinke et al., 2018) as well as in patient fibroblasts (Rodríguez et al., 2015; Viegas et al., 2022). NE proteins are linked to a wide range of disorders, including myopathies and neuropathies. Cellular functions of the NE include the organization, regulation, and repair of the genome, signaling, and cellular mechanics (Meinke and Schirmer, 2016). The composition of the NE is at least partially tissue specific (Korfali et al., 2012), and the identification of NE proteins in skeletal muscle (Wilkie et al., 2011) allows to investigate the NE role in DM1.

Here we reanalyzed RNA-sequencing data from deep sequencing of DM1 and control muscle biopsies (Wang et al., 2019) regarding muscle NE proteins to gain some insight in the role of the NE in DM1 and its contribution to the phenotype.

## Methods

### Sequencing data

The transcriptomes of 44 DM1 and 11 control tibialis biopsies are publicly available in FASTQ format at GEO (GSE86356). Sample processing has been described in (Wang et al., 2019). One DM1 sample was excluded from further analysis due to insufficient quality as assessed with fastqc. Anonymized patient information can be found in the supplementary data of the original publication and includes the evaluation of the normalized dorsiflexion strength in percent with healthy individuals corresponding to 100% of strength. For the subsequent analysis, either all samples or subgroups according to dorsiflexion strength were used. The subgroups are as following: healthy/proto DM1 (dorsiflexion strength 100%–75%), DM1 (dorsiflexion strength 75%–25%), and severe DM1 (dorsiflexion strength 25%–0%).

### Bioinformatical analyses

#### Alignment

Reads were either mapped with STAR v2.7 (Dobin et al., 2013) or Kallisto v0.46.0 (Bray et al., 2016) to the GRCh38 human reference genome. STAR generated BAM files were used for DESeq2 (Love et al., 2014), DEXSeq (Anders et al., 2012) and MAJIQ v2.3 (Vaquero-Garcia et al., 2016), Kallisto counts were used for isoformSwitchAnalyzer (Vitting-Seerup and Sandelin, 2019).

#### DESeq2

Aligned reads were counted using featureCounts and analyzed with a standard DESeq2 workflow in R v4.2 using the built-in normalization method (median of ratios). Principal component analysis (PCA) was used to plot the samples according to the two main parameters of variability PC1 and PC2, which showed that samples from healthy individuals clustered together, while DM1 patients are scattered along PC1, consistent with disease severity (Supplementary Figure S1). Genes with log2 foldchanges of > |0.5| and *p*-values < 0.05 have been set to be significantly changed. Gprofiler2 was used for GO analysis. Volcanoplots were generated with EnhancedVolcano, other plots have been generated with ggplot2. For the expression scatter plots of selected nuclear envelope transmembrane proteins in Figure 2 and Figure 3, samples were ordered after the normalized dorsiflexion strength. Additionally, the analysis has been run separately for the above determined three subgroups to find NE associated proteins. All results are in Supplementary Table S1.

#### DEXSeq

Mapped reads were counted using the in-built python script of DEXSeq with python v3.9. Standard DEXSeq workflow in R v4.2 was followed and exons with less than 40 counts for all samples filtered out. Exons with a log2FC of > |0.5| and *p*-value < 0.05 have been set to be significantly changed. Here as well, analysis has been run separately for the above determined three subgroups to find NE associated proteins. All results are in Supplementary Table S2.

#### Isoformswitchanalyzer

Isoform counts generated by Kallisto were imported in R and abundance values were normalized *via* edgeR. Normalized isoform expressions were used to generate bar charts *via* the in-built isoformSwitchAnalyzer function switchPlotIsoExp (). For this, we focused on the severe DM1 group and compared it to healthy controls. All results are in Supplementary Table S3.

#### MAJIQ

Alternative splicing events were analyzed using MAJIQ in python v2.7, providing STAR generated BAM files and a GRCh38 gff3 file. The in-built deltapsi script was used to determine significantly altered splice events between DM1 and control with a confidence interval of .9 and percent-spliced-in (psi) values of > |0.1|. MAJIQ Voila was used to visualize the splice graphs. Exon cassette results in Supplementary Table S4.

### Western blot

Whole protein extracts were generated from 10 µm muscle sections using RIPA buffer and an ultrasonic sonicator with a MS73 tip (Bandelin Sonopuls) to lyse the sections. The proteins were separated by SDS gel electrophoresis using 4%–15% TGX gels (BioRad #456–8,087) and 10% TGX gels (BioRad #456–8,034). Western blotting was performed using the Trans-Blot^®^ Turbo™ system (BioRad). Proteins were transferred to nitrocellulose membranes (Trans-Blot^®^ Turbo^™^ RTA Transfer Kit #170–4,270). Membranes were blocked with 5% skim milk in 1xTBS/0.1% Tween^®^ 20. Following primary antibodies were used: nesprin1 (provided by Didier Hodzic (Razafsky et al., 2013)), Tmem38a (Merck Millipore #06–1,005), Plpp7 (Proteintech #20635-1-AP). For quantification mouse antiGAPDH (Milipore #MAB374) was used. As secondary antibodies we used donkey anti-mouse IRDye 680RD and donkey anti-rabbit IRDye 800 CW. All western blot images were obtained using a Licor FC. Quantification was done using the Licor ImageStudio Software. Western blots were repeated at least three times to confirm the results. Full blots are shown in Supplementary Figure S2.

### Muscle biopsies

Muscle biopsies were obtained from the Muscle Tissue Culture Collection (MTCC) at the Friedrich-Baur-Institute (Department of Neurology, LMU Klinikum, Ludwig-Maximilians-University, Munich, Germany). All materials were obtained with written informed consent of the donor. Ethical approval for this study was obtained from the ethical review committee at the Ludwig-Maximilians-University, Munich, Germany (reference 45–14).

## Results

To analyze muscle NE protein expression and splicing in DM1 biopsies we used a list of 386 proteins identified by mass spectrometry of isolated muscle NEs (Wilkie et al., 2011; Korfali et al., 2012) (**Supplemental Table S5**). The genes encoding these 386 proteins were analyzed for alterations in expression or splicing in a published transcriptome dataset of 54 tibialis anterior muscle biopsies (Wang et al., 2019). We decided to use the datasets of tibialis anterior muscles for our analyses as this muscle is predominantly affected in DM1 (Harper, 2001). These 54 tibialis anterior muscle biopsies originated from 11 unaffected individuals and 43 DM1 patients, all characterized for ankle dorsiflexion strength to quantify how much the muscle was affected. Based on these measurements the DM1 patients were characterized as proto-DM1, DM1 or severe DM1 (Wang et al., 2019).

### Differential expression of muscle nuclear envelope proteins

**FIGURE 1:**
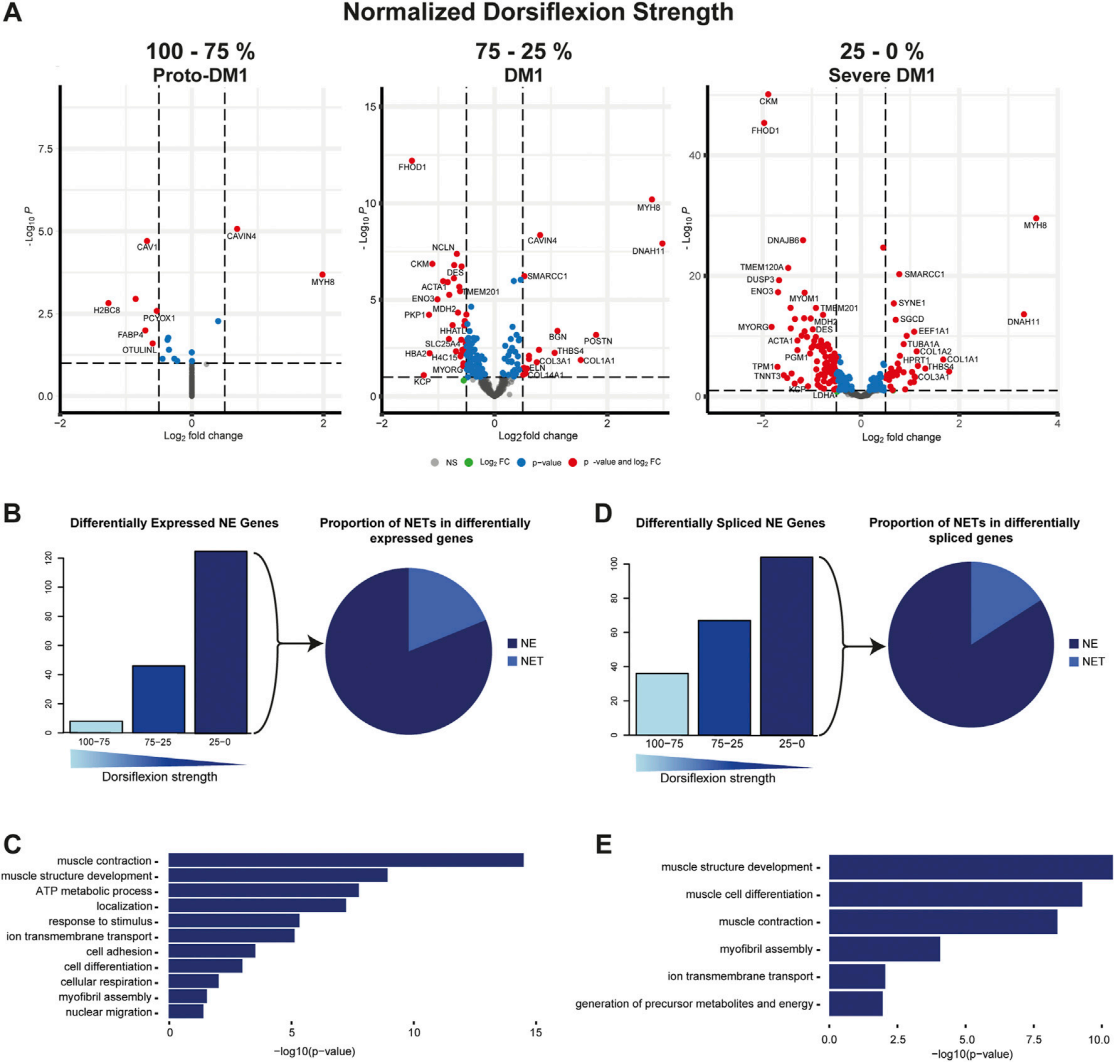
Muscle nuclear envelope (NE) proteins are differential expressed and spliced in DM1. **(A)** Gene expression of 386 muscle NE proteins for proto-DM1, DM1, and severe DM1 based on dorsiflexion strength. **(B)** Number of genes being differential expressed and the proportion of NE transmembrane proteins (NETs). **(C)** GO-term enrichment for the differential expressed muscle NE proteins. **(D)** Number of genes being differential spliced and the proportion of NETs. **(E)** GO-term enrichment for the differential spliced muscle NE proteins.

First, read counts were analyzed using DEseq2. We identified two genes up and six genes being downregulated in proto-DM1, while in DM1 14 genes were up and 32 genes downregulated. In severe DM1, there was a further increase of NE-protein encoding genes being differentially expressed, 34 genes were up and 91 genes down (Figure 1A). The total number of genes encoding muscle NE proteins was accordingly increasing with loss of dorsiflexion strength (8, 46, 125; Figure 1B, left panel). Among these differentially expressed genes, the percentage of genes encoding proteins with a transmembrane domain was 11.2% (Figure 1B, right panel). Next, we were interested in which biological functions the protein products of these genes were involved. Pathway analysis revealed functions in muscle relevant processes like muscle contraction, muscle structure development, response to stimulus, and metabolic processes (Figure 1C; Supplementary Table S6).

### Splicing alterations of muscle nuclear envelope proteins

Apart from differential expression, splicing alteration can impact the function of translated proteins—especially when considering that the main pathomechanism described in DM1 is an increase in alternative splicing. Similar to differential gene expression the number of genes affected by splice alterations did increase with reduced dorsiflexion strength. In proto-DM1, 36 genes were affected while in DM1 this number increased to 67 and in severe DM1 to 104 genes (Figure 1D, left panel). Among these differentially spliced genes, the percentage of genes encoding proteins with a transmembrane domain was 8.7% (Figure 1D, right panel). Pathway analysis of these alternatively spliced genes also revealed functions in muscle contraction, muscle structure development, and metabolic processes (Figure 1E; Supplementary Table S7).

### Cytoskeletal associated NETs

**FIGURE 2:**
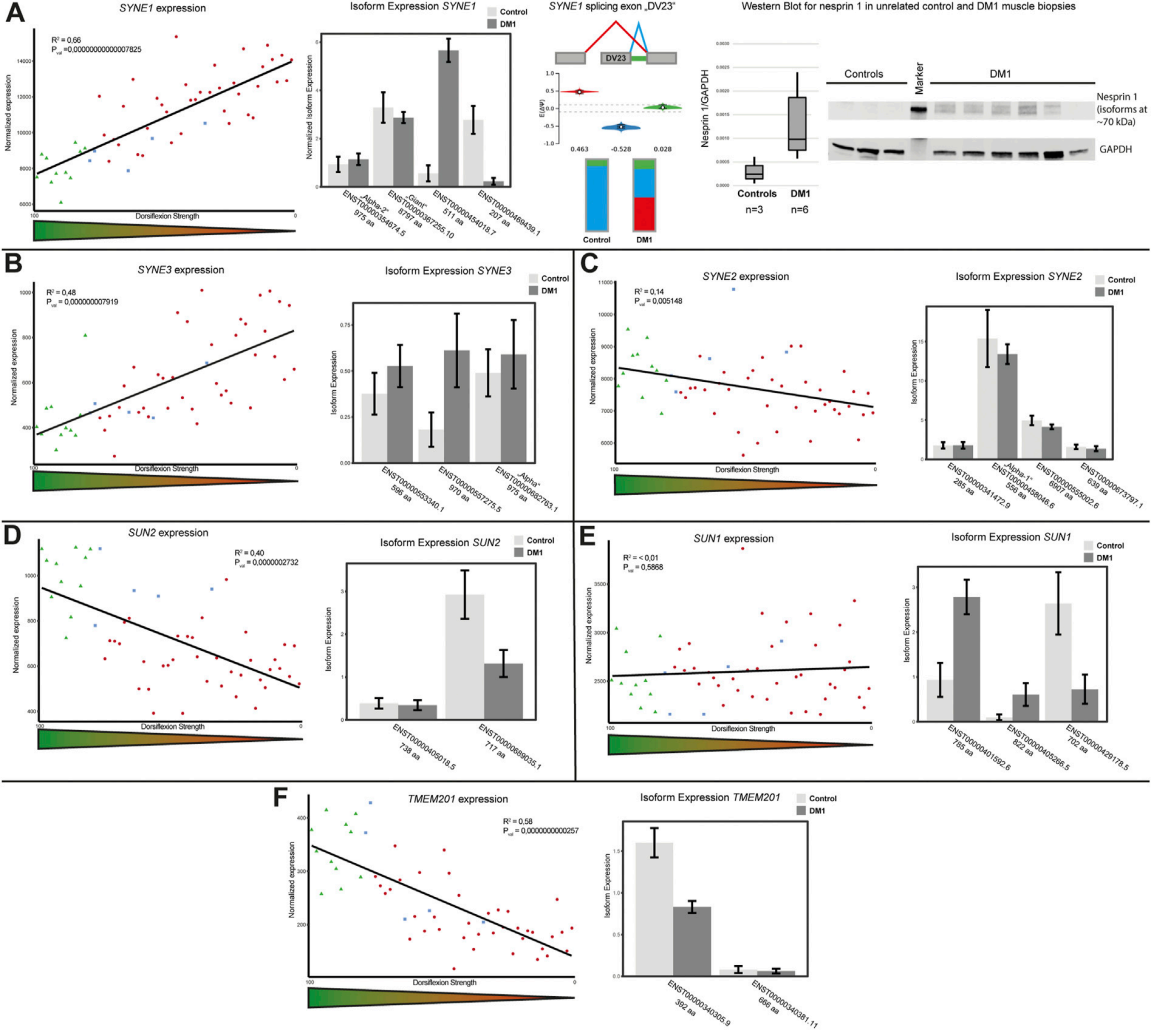
LINC (linker of nucleo- and cytoskeleton) complex protein expression and splicing is altered in DM1. **(A)** *SYNE1* gene expression is inversely correlated with dorsiflexion strength (left panel), there is a switch in isoform expression (second panel) and the muscle-specific DV23 exon is preferentially spliced out in DM1 (third panel). Western blot analysis confirms the expression changes for short nesprin isoforms (fourth panel). **(B)** *SYNE3* gene expression is inversely correlated with dorsiflexion strength (left panel), there is a switch in isoform expression (right panel). **(C)** *SYNE2* gene expression slightly correlates with dorsiflexion strength (left panel), there appears to be a general slight downregulation of several isoforms (right panel). **(D)** *SUN2* gene expression correlates with dorsiflexion strength (left panel), the main muscle isoform is downregulated (right panel). **(E)** *SUN1* gene expression is unaffected (left panel), but there is a switch in isoform expression (right panel). **(F)** *TMEM201* gene expression correlates with dorsiflexion strength (left panel), the main muscle isoform is downregulated (right panel).

We did describe alterations of the NE in primary DM1 myoblasts and myotubes before (Hintze et al., 2018; Meinke et al., 2018). There, we observed NE invaginations which indicated altered nuclear-cytoskeletal coupling and accordingly identified altered expression of several nesprin isoforms. Based on these data we screened differentially expressed and spliced genes for genes encoding LINC complex (linker of nucleo- and cytoskeleton) and LINC-associated proteins. We identified the expression of *SYNE1*, encoding nesprin 1, to be inverse correlated with dorsiflexion strength (Figure 2A, left panel). As the *SYNE1* gene is giving rise to multiple nesprin isoforms by alternative splicing, we performed an isoform expression analysis. This showed that the expression changes were not caused by alterations of the giant or muscle specific alpha-2 isoforms. Instead, there was a misregulation of other short isoforms, as illustrated for two isoforms containing neither the KASH nor the actin-binding domain. While a 207 amino acid (aa) isoform was downregulated a 511 aa isoform was strongly upregulated (Figure 2A, second panel). The *SYNE1* gene also came up in the MAJIQ analysis, with a preferential exclusion of a specific exon. This 69 nucleotide exon was identified in an early study (Apel et al., 2000) and later named ΔSR (Simpson and Roberts, 2008) and DV23 (Duong et al., 2014). It is evolutionary conserved and highly muscle-specific (Simpson and Roberts, 2008; Duong et al., 2014). We found this exon to be spliced out in about 50% of the transcripts in DM1 biopsies while it was almost exclusively spliced in in controls (Figure 2A, third panel). To verify the RNAseq data on protein level we performed Western blot on a set of unrelated control and DM1 muscle biopsies. An increased signal of several bands between 70 and 260 kDa in DM1 patients muscle indicates an upregulation of short nesprin 1 isoforms on protein level (Figure 2A, fourth panel; Supplementary Figure S2).

Similar to *SYNE1* the expression of *SYNE3*, encoding nesprin 3, was also inverse correlated with dorsiflexion strength (Figure 2B, left panel). Here the increased expression appears to originate from an upregulation of a 970 aa isoform, which differs from the “alpha isoform” (975 aa) by the loss of the amino acids 793 to 797 due to the usage of an alternative splice site (Figure 2B, right panel). Considering the differential expression of the nesprins 1 and 3 we decided to look also at *SYNE2*, but here we found only a very mild trend for a correlation of gene expression and dorsiflexion strength which may be caused by changes to the expression of the muscle isoform “alpha-1” (Figure 2C).

The nuclear side of the LINC complex consists of SUN proteins. The expression of *SUN2* was strongly correlated with dorsiflexion strength (Figure 2D, left panel). In terms of isoform expression, this seems to originate from a downregulation of the 717 aa isoform (Figure 2D, right panel). We also looked at expression of *SUN1*, but could not find clear correlation with dorsiflexion strength (Figure 2E, left panel). However, looking at the isoform expression we could see several alterations which seem to level out the total gene expression. While a 785 aa and a 822 aa isoform were upregulated, a 702 aa isoform was strongly downregulated (Figure 2E, right panel).

Samp1, which is encoded by the *TMEM201* gene, is functional associated to the LINC complex (Gudise et al., 2011). We found expression of Samp1 to strongly correlate with dorsiflexion strength (Figure 2F, left panel). The reduced expression is due to downregulation of the shorter isoform (392 aa), with the longer isoform (666 aa) being affected very little (Figure 2F, right panel).

### Genome organizing mNETs

Samp1 has not only been described to be involved in the nucleo-cytoskeletal coupling *via* the LINC complex, but has also been shown to be involved in genome organization (Zuleger et al., 2013). This in addition to observed general gene expression changes here as well as in DM1 tissue culture systems (Todorow et al., 2021) prompted us to investigate muscle specific NETs involved in genome organization in more detail. We found in addition to *TMEM201* the expression of *PLPP7*, *TMEM38A*, *TOR1AIP1* and *EMD* to be altered.

**FIGURE 3:**
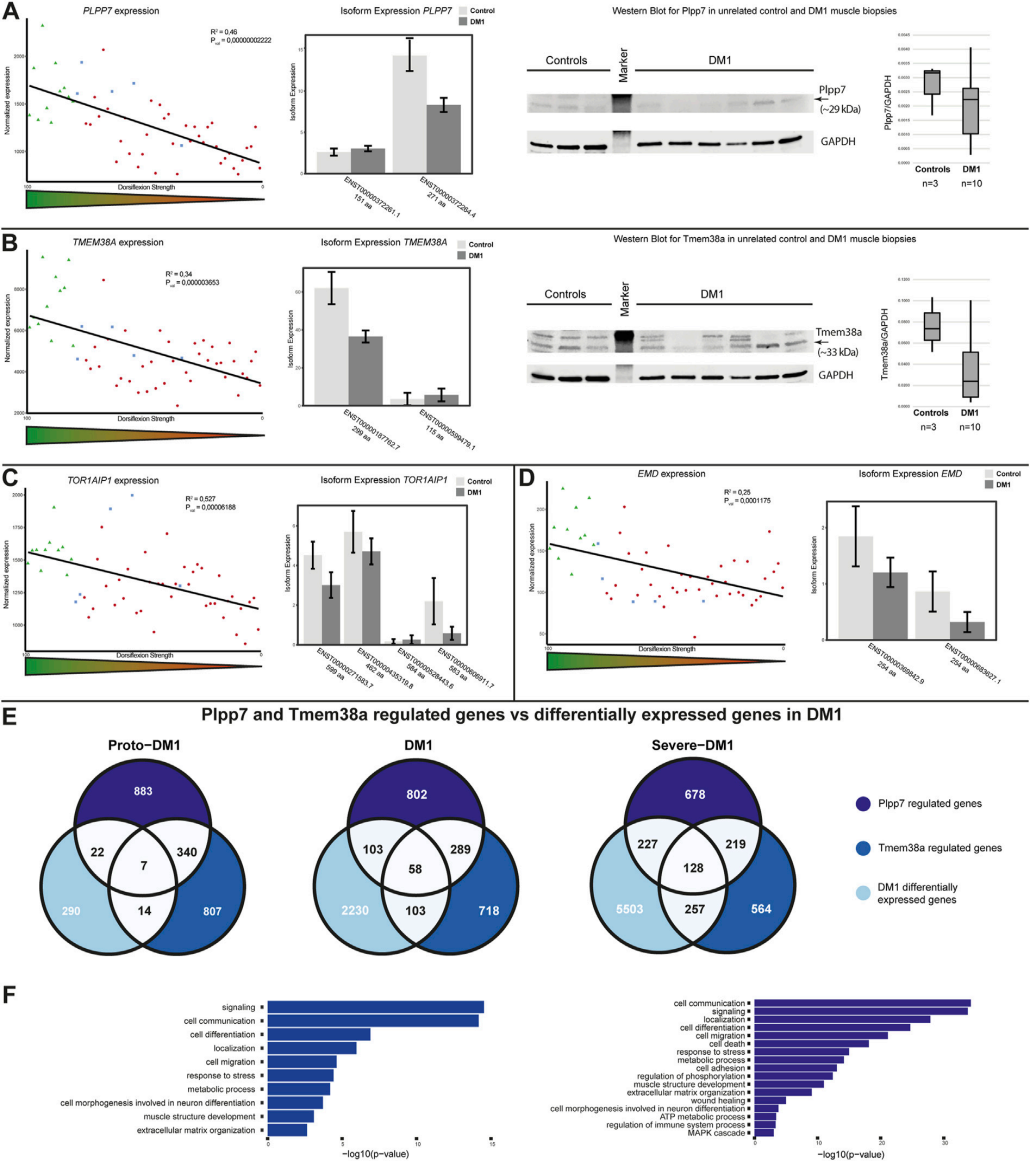
Genome organizing muscle nuclear envelope transmembrane protein (NET) expression is altered in DM1 and affects gene expression. **(A)** *PLPP7* gene expression is correlated with dorsiflexion strength (left panel), the main muscle isoform is downregulated (middle panel). Western blot analysis confirms the expression changes (right panel). **(B)** *TMEM38A* gene expression is correlated with dorsiflexion strength (left panel), the main muscle isoform is downregulated (middle panel). Western blot analysis confirms the expression changes (right panel). **(C)** *TOR1AIP1* gene expression is correlated with dorsiflexion strength (left panel), several isoforms are downregulated (right panel). **(D)** *EMD* gene expression is correlated with dorsiflexion strength (left panel), several isoforms only different in the UTR-region are downregulated (right panel). **(E)** Overlap between Plpp7 and Tmem38a regulated genes and DM1 differential expressed genes for proto-DM1, DM1, and severe DM1 based on dorsiflexion strength. **(F)** GO-term enrichment for the Plpp7 and Tmem38a regulated and DM1 differential expressed genes.

For *PLPP7* we found a positive correlation of gene expression and dorsiflexion strength (Figure 3A, left panel). This was caused by downregulation of the main isoform of the protein (271 aa) (Figure 3A, middle panel). We proceeded to confirm these expression changes on protein level by Western blot, which showed downregulation of Plpp7 in unrelated DM1 muscle biopsies (Figure 3A, right panel). Tmem38a expression was correlating in a similar manner as *PLPP7* with dorsiflexion strength (Figure 3B, left panel). Here the expression changes also seemingly originated from the main isoform (299 aa) (Figure 3B, middle panel). We could also confirm these results on protein level in unrelated samples (Figure 3B, right panel). We looked at two additional NETs known to be involved in genome organization, LAP1 (encoded by *TOR1AIP1*), and emerin (encoded by *EMD*). For both we found a clear correlation with dorsiflexion strength originating from a downregulation of all isoforms (Figures 3C, D).

Apart from the effect on expression and splicing of muscle NE proteins we were also interested in possible functional consequences. Plpp7 and Tmem38a are muscle specific NETs involved in genome organization, and the genes they contribute to regulate in C2C12 myotubes (which partially overlap) have been identified (Robson et al., 2016). To investigate whether the observed expression changes in DM1 muscle biopsies do have any functional relevance we proceeded to test the expression of these Plpp7 and Tmem38a co-regulated genes in the three subgroups. We could indeed find an overlap between genes regulated by both proteins in mouse myotubes and DM1 patient biopsies: in proto-DM1, there was an overlap of 43 genes, in DM1 264 genes, and in severe DM1 612 genes (Figure 3E). This made up 13, 11, and 10% of the overall differentially expressed genes in the DM1 samples, respectively. Next, we were interested in the biological functions of the genes under Plpp7 or Tmem38a control. Considering the number of genes, this analysis was possible for the DM1 and severe DM1 groups. The main enriched pathways were signaling, cell communication, cell migration, localization, response to stress and metabolic process (Figure 3F, Supplementary Table S8).

## Discussion

The missplicing in DM1 is well investigated and there are many target genes of this missplicing described, which are contributing or likely contributing to the disease pathology. Yet, it still remains elusive which additional pathomechanisms are contributing to the development in DM1, and to which extent, especially in the different tissues affected. The NE has been shown to be much more than just a barrier separating the genome from the rest of the cell (de Las Heras et al., 2013), it hosts a tissue specific proteome and tissue specific as well as ubiquitously expressed NETs have been shown to be involved in controlling the intranuclear positioning and thus expression of genes, often in a tissue specific manner (Zuleger et al., 2011). We could previously identify NE alterations in muscle tissue culture systems of DM1, with likely effects on cell cycle control and differentiation (Hintze et al., 2018; Meinke et al., 2018). Investigating the involvement of the NE in DM1 mature muscle was therefore the logical follow up to unravel its role in the DM1 pathology.

The set of NE genes we investigated contained genes with and without transmembrane domains, as we did not want to exclude a possible contribution of NE-associated proteins. We could indeed find for both NE and NET encoding genes a high percentage of differential expression and differential splicing. This highlighted the likelihood of an important role of NE proteins in DM1 as the GO-term analysis revealed that the most enriched processes of these differentially regulated genes are all relevant for muscle function.

We wanted to follow up on specific aspects of NE function. Considering the misregulation of nesprin proteins in DM1 muscle cell cultures (Hintze et al., 2018), which is a possible explanation for observed NE invaginations (Meinke et al., 2018), and the identification of mutations in *SYNE* and *SUN* in a clinically similar disease, EDMD (Zhang et al., 2007; Meinke et al., 2014), we looked at all components of the LINC complex. We could identify isoform-specific alterations in the expression of the *SYNE1*, *SYNE3*, *SUN1*, and *SUN2* genes—all core components of LINC complexes. Although there was no apparent change in the expression of the muscle-specific nesprin 1 isoform “alpha-2”, in about half of these transcripts a 23 aa exon (DV23) was spliced out in DM1 patients. As this exon has been shown to be included in 94% muscle *SYNE1* transcripts (Duong et al., 2014) this could indicate a loss of a muscle-specific nesprin 1 function. Furthermore, for Samp1, which has been identified as a LINC complex associated protein, there was also a downregulation of the major muscle isoform. This clearly indicates a likely weakening of the nuclear-cytoskeletal connection in DM1 muscle, which is going to impact on mechanotransduction as well as nuclei positioning. This is in line with observed missplicing of the myc box-dependent-interacting protein 1 (Bin1), which is involved in the formation of tubular invaginations of the plasma membrane that function in depolarization-contraction coupling (Fugier et al., 2011).

Another important aspect of the NE is the tissue specific regulation of gene expression by the recruitment of specific genes to the NE by tissue specific NETs. Examples for this are Plpp7 and Tmem38a, which have been shown to have important muscle functions (Robson et al., 2016). It is important to note that these proteins appear to have an additive effect, a knockdown of more than one resulted in stronger effects than single knockdowns (Robson et al., 2016). Thus, it is likely that a reduced expression of several NETs is also adding up to result in phenotypical consequences. We found both proteins to be downregulated on RNA and protein level, and by comparing DM1 differentially expressed genes to the genes identified under their expression control in mouse myoblasts, we could prove that we have similar effects in DM1. Intriguingly, mutations in *PLPP7*, *TMEM38A*, and *TMEM201* have been identified in muscular dystrophy patients with an EDMD-like phenotype (Meinke et al., 2020), which highlights the importance of these proteins in muscle disease. Misregulation of the two muscle NETs Plpp7 and Tmem38a alone does indeed account for about 10% of all differentially expressed genes in DM1 muscle. Since there are additional NETs misregulated in DM1 the actual effect is probably even more profound. It has been shown that Samp1 can also reposition chromosomes (Zuleger et al., 2013) and emerin directly binds histone deacetylase 3 (Demmerle et al., 2012), while LAP1 binds indirectly to chromatin (Foisner and Gerace, 1993). Notably, mutations in the genes encoding emerin and LAP1 also cause EDMD (Bione et al., 1994) respectively a very similar muscular dystrophy (Kayman-Kurekci et al., 2014). All in all, our data suggests that DM1 and EDMD share a broader common ground also on the cellular level rather than only in the symptomology.

## Data Availability

The datasets presented in this study can be found in online repositories. The names of the repository/repositories and accession number(s) can be found in the article/Supplementary Material.
