# Supplementary figures and images for: Nuclear envelope transmembrane proteins involved in genome organization are misregulated in myotonic dystrophy type 1 muscle
Source: Front Cell Dev Biol. 2023 Jan 9;10:1007331. doi: 10.3389/fcell.2022.1007331 (PMC9868253; doi:10.3389/fcell.2022.1007331)

IRDye 680RD (red)

kDa

IRDye 800 CW (green)

GAPDH

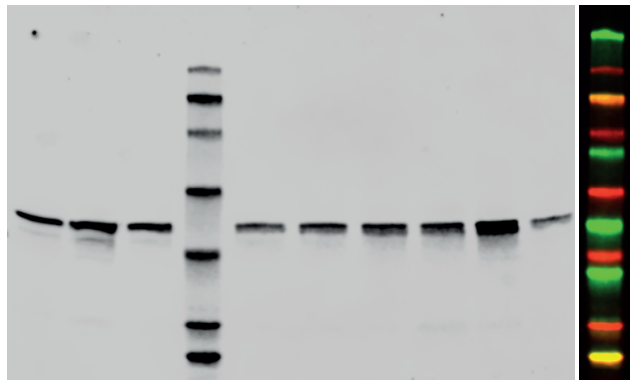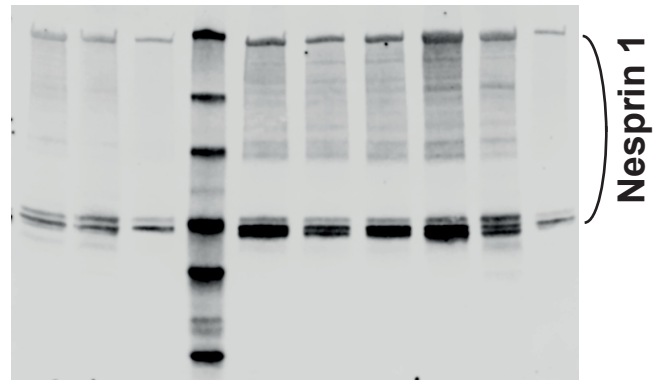

GAPDH

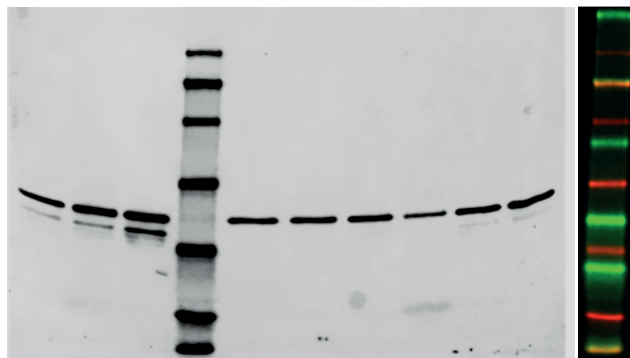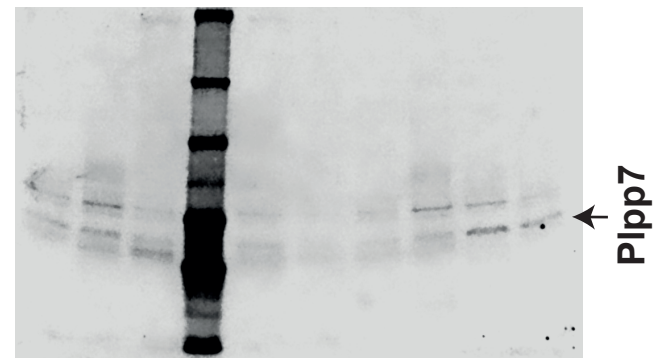

GAPDH

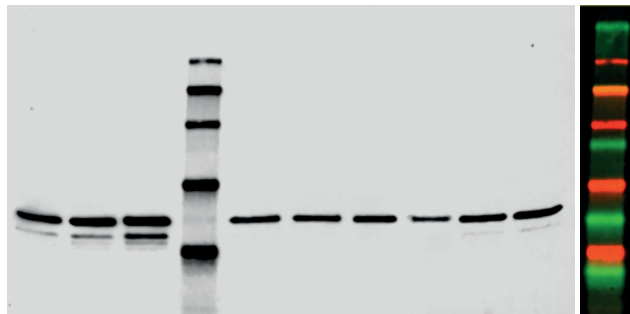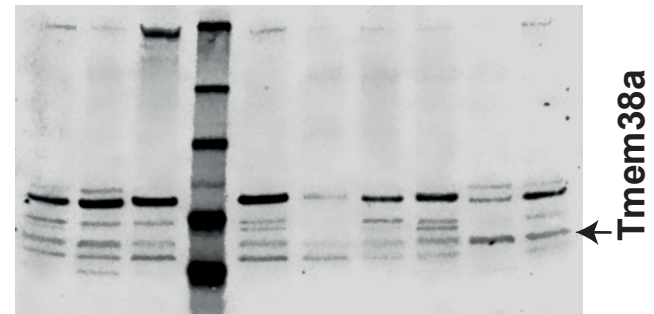

Chameleon® Duo Pre-stained Protein Ladder

Supplement: Supplementary file 1 [file DataSheet2.PDF]

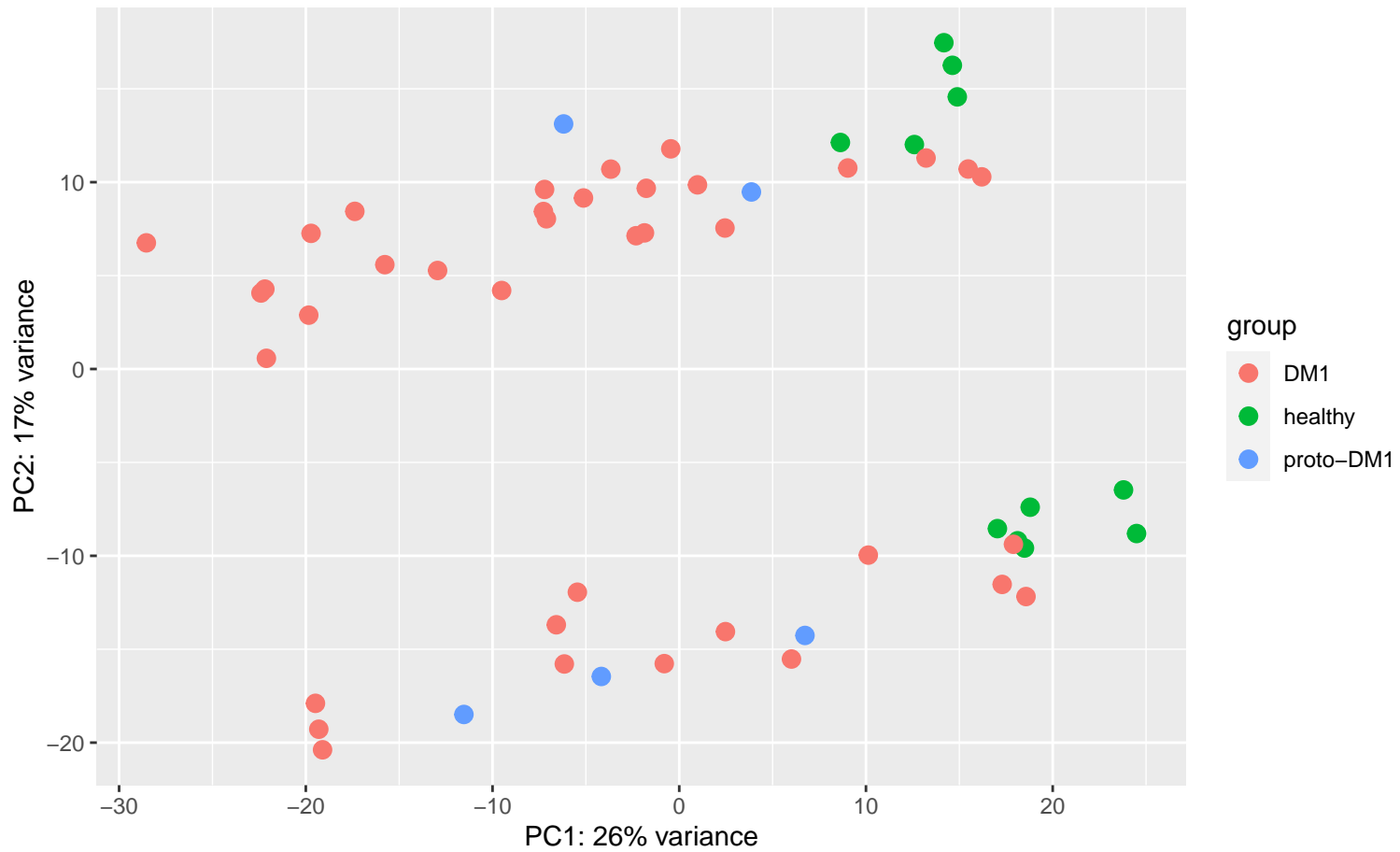

Supplement: Supplementary file 5 [file DataSheet1.PDF]
